# Fluoride-related changes in the fetal cord blood proteome; a pilot study

**DOI:** 10.21203/rs.3.rs-3995767/v1

**Published:** 2024-03-01

**Authors:** Sami T. Tuomivaara, Dana E. Goin, Susan J. Fisher, Steven C. Hall, Aras N. Mattis, Pamela K. Den Besten

**Affiliations:** University of California, San Francisco San Francisco; University of California, San Francisco San Francisco; University of California, San Francisco San Francisco; University of California, San Francisco San Francisco; University of California, San Francisco San Francisco; University of California, San Francisco San Francisco

## Abstract

**Background:**

Fluoride exposure during pregnancy has been associated with various effects on offspring, including changes in behavior and IQ. To provide clues to possible mechanisms by which fluoride affects human fetal development, we completed proteomic analyses of cord blood serum collected from second-trimester pregnant women residing in Northern California with either high or low fluoride exposure, as identified by maternal serum fluoride concentrations.

**Objective:**

To identify changes in cord blood proteins associated with maternal serum fluoride concentration in pregnant women living in Northern California.

**Methods:**

The proteomes of 19 archived second-trimester cord blood samples representing highest and lowest serum fluoride concentrations from a cohort of 48 women living in Northern California, previously analyzed for serum, urine and amniotic fluoride concentrations, were characterized by mass spectrometry. Proteins highly correlated to maternal serum fluoride concentrations were identified, and further compared in a group of samples from women with the highest serum fluoride to the group with the lowest maternal serum fluoride concentrations.

**Results:**

Nine cord blood proteins were significantly correlated with maternal serum fluoride concentrations. Six of these proteins, including apolipoprotein B-100, delta homolog 1, coagulation factor X, mimecan, plasma kallikrein, and vasorin, were significantly decreased in the cord blood from women with the highest serum fluoride levels.

**Conclusion:**

Changes in the relative amounts of second trimester cord blood proteins included proteins associated with the development of the fetal hematopoetic system.

## INTRODUCTION

Tooth enamel fluorosis, a biomarker for fluoride exposure, has steadily increased in the US [1, 2] resulting in hypomineralization of tooth enamel and at higher exposures, increasing the risk for dental disease[3]. Increasing levels of fluorosis are of concern in view of recent human studies showing associations between fluoride exposure during either pregnancy or the first year of life, and impaired neurodevelopment of offspring [4–9].

Rodent models show fluoride-related effects on cell function and neurodevelopment [10, 11], associated with increased oxidative stress and inflammation [12–16]. However, the relevance of these studies to human health has been questioned because rodents require 5 to 10-fold higher levels of fluoride in drinking water to achieve plasma fluoride levels similar to those found in humans [17]. Mechanisms responsible for possible fluoride-related effects in humans remain poorly understood, though reports of age and sex related effect of fluoride on children, identified by analyses of NHANES data, suggest that fluoride can affect multiple cells and tissues[18–22].

In this study, we investigated possible effects of fluoride on the developing fetus by comparing the cord blood proteome of second-trimester pregnant women in Northern California [23]. Cord blood samples were obtained from a random cohort of 48 samples of healthy second trimester pregnant women that we had previously analyzed for serum, urine, and amniotic fluid fluoride concentrations. Urine fluoride concentrations of this cohort were similar to cohorts from larger retrospective studies of Candian [23] and Mexican women [5, 6] showing fluoride related change in offspring neurodevelopment.

## METHODS

### Study Samples

This study was approved by the University of California, San Francisco Committee on Human Research. Archived cord blood serum samples, from a cohort of 48 women [23], were selected for whole proteome analysis. Ten samples were from women who had the lowest serum fluoride, and 9 samples were from women with the highest serum fluoride in from this cohort. All women resided in communities with water fluoride levels ranging from 0.2 to 0.9 ppm. The community water fluoride concentration recommended by CDC was 1.0 ppm prior to 2015, and was subsequently change to 0.7 ppm fluoride in 2015, and therefore all were near or below the maxium recommended levels for artificial water fluoridation.

### Proteomic analysis

The cord blood samples were doped with protease inhibitors, and after clearing and dilution, the 14 most abundant proteins, including serum albumins were removed using immobilized antibodies. Total protein concentration was determined with BCA protein assay. The samples were buffer exchanged into ammonium bicarbonate, denatured with urea, reduced and alkylated, and digested with trypsin. The resulting peptide mixtures were desalted and analyzed by liquid chromatography-tandem mass spectrometry. Protein abundance in a sample was inferred from the number of peptide identifications. To account for the analytical variability, relative peptide abundance was calculated for each protein in each sample by dividing the number of peptides identified from a given protein by the total number of peptides identified in that sample.

### Statistical analyses

Demographics were compared by Kruskal-Wallis one-way analysis of variance test. Pearson’s correlation coefficients were calculated for the relative protein abundance. The samples were grouped relative to highest and lowest maternal serum fluoride concentrations and and significance (*p*-value) from paired Welch’s t-test were calculated. All data analyses were conducted in the R statistical computing environment (version 4.0 or newer).

## RESULTS

Demographics of the study population showed community water fluoride levels at the time of sample collection from the 10 women with the lowest serum fluoride concentrations (0.0040 to 0.0073 ppm) ranged from 0.2 to 0.8 ppm. Community water levels at the time of sample collection, for 9 women with higher serum fluoride (0.0224 to 0.0593 ppm fluoride) ranged from 0.16 to 0.9 ppm. Community water fluoride concentrations for all subjects were near or below 1.0 ppm, which was the optimal water fluoride levels recommended by the Center for Disease Control (CDC) at the time of collection.

The group of women with higher serum fluoride levels, were further divided into middle range serum fluoride of 0.0224 to 0.0291 ppm fluoride, and high serum fluoride ranging from 0.0444 to 0.0593 ppm fluoride. There were no significant differences in maternal age, smoking status or ethnicity between the fluoride groups (Table 1). However, the higher community water fluoride levels were associated with higher serum fluoride concentrations, with significant differences between groups.

**Figure 1:**
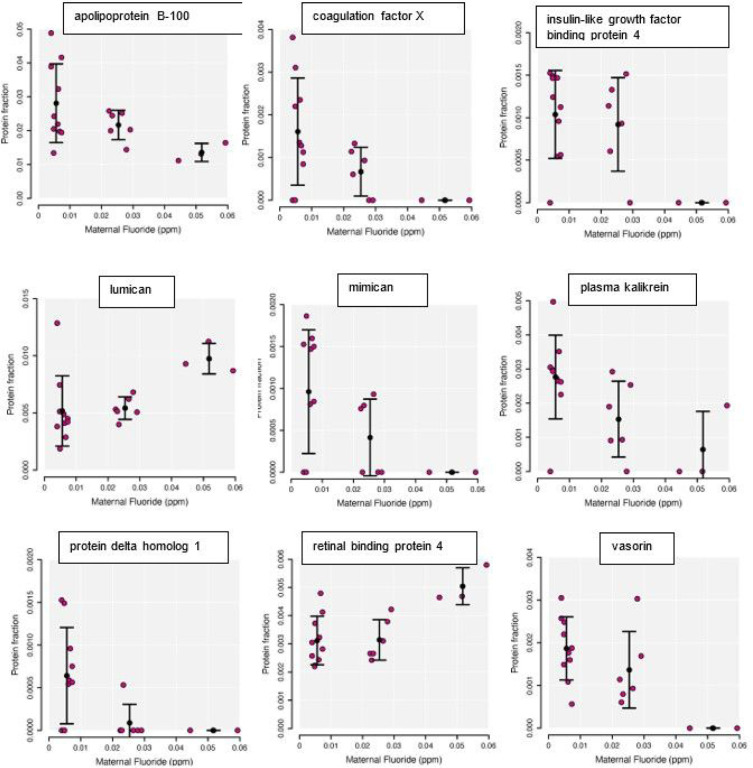
Plots showing fetal cord blood proteins significantly correlated to maternal serum fluoride concentrations. Relative amounts of protein per fraction for individual samples at each maternal serum fluoride concentration, are shown by the red dots. Standard deviations for low, medium and high maternal serum fluoride, are shown by bars.

Mass spectrometry analysis of the cord blood proteome of the 19 samples showed significant correlations (Pearson’s correlation coefficient greater or less than 0.5) of 9 proteins with maternal serum fluoride concentrations (**see** Fig. 1).

Proteins identified as significantly correlated to maternal serum fluoride were grouped into low and higher serum fluoride concentration (combined medium and high serum fluoride) and relative protein concentrations were compared. Six of the 9 cord blood proteins were significantly different in the low as compared to higher fluoride group (p ≤ 0.05) (see Table 2). These cord blood proteins were all significantly reduced in the higher as compared to lower fluoride group.

## DISCUSSION

Fetal circulation occurs through the umbilical vein that carries maternal blood and nutrients filtered through the placenta to the fetus, and 2 umbilical arteries that transport blood to and from the placenta. The placenta forms a barrier to macromolecules, including most proteins, to cross from maternal to fetal circulation [30]. However, fluoride crosses the placenta [23, 31], and is also increased in the placenta relative to cord blood fluoride concentrations [32, 33]. Previous studies have shown that fetal cord blood fluoride concentrations reflect maternal blood fluoride [34], and therefore in this study we used maternal serum fluoride concentrations as a biomarker for fetal fluoride exposure [23].

Cord blood serum was collected between 2014 and 2016 from second trimester pregnant women residing in Northern California [23]. All women were healthy without known underlying medical conditions, and their community water fluoride concentrations were at or below optimal water fluoride concentrations, as recommended by the US Center for Disease Control (CDC) at the time of sample collection (1.0 ppm fluoride). The 9 cord blood proteins found to be significantly associated with the maternal serum fluoride concentration were not correlated with risk factors for pregnancy outcomes including maternal age [35, 36], smoking status [37, 38], BMI [39], and race/ethnicity.

These proteins included protein delta-like homolog 1 (DLK1), a transmembrane protein highly expressed by stromal cells of the placental villi [28] and by the fetal liver hepatocytes [40], which was negatively associated with maternal serum fluoride. DLK1 regulates glucose metabolism [41] and placental growth hormone [42], and is reduced in small for gestational age fetuses [28]. The placenta is a primary hematopoietic stem cell (HSC) niche during pregnancy, which is believed to seed the fetal liver [43, 44], and depletion of DLK1 from of human fetal liver hepatoblasts in culture reduces the percentage of mature hematopoietic cells [40].

DLK1 also regulates notch signaling and affects neurodevelopment [45]. In mice, DLK1 was shown to regulate hippocampal neurogenesis and cognition. Reduced DLK1 levels triggers cognitive abnormalities [46], suggesting the possibility that fluoride-related effects on fetal neurodevelopment may be associated with reductions in DLK1.

The fetal liver is the primary organ for erythropoiesis during development, functioning as a vascular connection between the developing placental vessels connecting the heart [47]. It does not perform the traditional digestive and filtration functions because nutrients are normally carried to the fetus from the mother via the placenta. Other proteins significantly downregulated relative to maternal serum fluoride are also associated with hematopoiesis, and include apolipoprotein B-100 (APOB), coagulation factor X, plasma kallikrein, and vasorin. APOB, which is synthesized by the placenta, is a likely pathway for lipid transfer required for fetal growth [24]. Vasorin is a transmembrane glycoprotein[48] with a possible role in the regulation of the glycogen-mediated mTOR-ULK1 signaling pathway in the liver[49], and mimecan, also known as osteoglycin which is a small leucine-rich proteoglycan (SLRPs) present in the extracellular matrix of multiple tissues, including adipose tissue.

The possibility that fluoride may affect the liver in humans is supported by NHANES survey data from 2013–2016 that shows higher water fluoride and plasma fluoride concentrations are associated with lower blood urea nitrogen among US adolescents. However, while the fetal hematopoietic system is separate from that of the maternal circulatory system, the placenta is of dual origin, comprised of both fetally and maternally derived cells and maternal inflammation can alter maternal–fetal interactions. Maternal immune activation during pregnancy, can affect the development of the fetal hematopoietic system [50], and therefore it possible that fluoride related effects to increase maternal inflammation may also have a role in fetal development. Analysis of available NHANES survey data (2013–2014 and 2015–2016) of children aged 6–19, shows plasma fluoride concentrations positively correlated with total white blood cell count, segmented neutrophils and monocytes when adjusted for age, gender, and BMI as predictors. While there is no available data linking second trimester maternal white blood cell counts to fluoride exposure, further studies to explore this possibility are warrented.

In summary, this is the first study to assess possible fluoride related changes associated with human fetal development at low level fluoride exposure. Our finding in this pilot study, that cord blood proteins associated with the development of the fetal hematopoietic system were significantly downregulated relative to maternal fluoride exposure, supports the possibility that fluoride related mechansims affecting fetal development and may be associated with the placenta and fetal hematopoesis during second trimester fetal development. The second trimester of fetal development is also critical for development of the prefrontal cortex and integration of control of attention, thought, emotions and actions [51]. Therefore, the identification of cord blood proteins altered by relative levels of maternal fluoride exposure may direct future studies of fluoride related mechanism that may affect fetal development, including neurodevelopment.

## Figures and Tables

**Table 1:** Demographics of study population (N = 19)

	Low serum fluoride	Middle serum fluoride	High serum fluoride	p-value
N	10	6	3	
Mean (SD)				
Serum fluoride (ppm)	0.006 (0.001)	0.025 (0.003)	0.052 (0.007)	0.00
Water fluoride (ppm)	0.36 (0.32)	0.59 (0.26)	0.80 (0.11)	0.05
Age	24.0 (3.9)	23.8 (3.5)	25.3 (4.6)	0.82
BMI	27.3 (5.8)	25.5 (3.4)	27.5 (3.5)	0.80
N (%)				
Smoker	6 (60.0)	4 (66.7)	3 (100.0)	0.44
Race/ethnicity				
White	5 (50.0)	3 (50.0)	2 (66.7)	0.88
Black	3 (30.0)	2 (33.3)	1 (33.3)	0.99
Latina	2 (20.0)	1 (16.7)	0 (0.0)	0.72

**Table 2.** 

Protein (gene)	Pearson’s correlation coefficient n = 19	Higher (n = 9) vs low (n = 10) F (paired Welsh’s t-test)	Fetal Tissue	Biological process
Apolipoprotein B-100	−0.532	p = 0.045*	Placenta liver	Transports lipids from mother to fetus[24]
Coagulation Factor X	−0.571	p = 0.020*	Liver uterine wall	Coagulation and wound healing [25]
Mimecan	−0.531	p = 0.024*	Extracellular matrix of multiple types of cells and tissues	Regulation of glucose metabolism; negatively correlates with fat and bone mass [26]
Plasma Kallikrein	−0.554	p = 0.012*	Blood vessel walls	Coagulation and wound healing [27]
Protein delta homolog 1	−0.527	p = 0.010*	Placenta	Placental growth [28]
Vasorin	−0.656	p = 0.031*	Liver; vascular, and smooth muscle	Blood vessel contraction [29]

## Data Availability

The datasets generated and/or analyzed during the current study are not publicly available due to patient privacy protections, but are available from the corresponding author on reasonable request.

## References

[1] Dong H, Yang X, Zhang S, Wang X, Guo C, Zhang X, Ma J, Niu P Chen T: Associations of low level of fluoride exposure with dental fluorosis among U.S. children and adolescents, NHANES 2015–2016. Ecotoxicology and Environmental Safety 2021, 221:112439. 34166938 10.1016/j.ecoenv.2021.112439

[2] Neurath C, Limeback H, Osmunson B, Connett M, Kanter V, Wells CR: Dental Fluorosis Trends in US Oral Health Surveys: 1986 to 2012. JDR Clin Trans Res 2019, 4(4):298–308. 30931722 10.1177/2380084419830957

[3] Marín LM, Cury JA, Tenuta LMA, Castellanos JE, Martignon S: Higher Fluorosis Severity Makes Enamel Less Resistant to Demineralization. Caries Research 2016, 50(4):407–413. 27458817 10.1159/000447270

[4] Choi AL, Zhang Y, Sun G, Bellinger DC, Wang K, Yang XJ, Li JS, Zheng Q, Fu Y, Grandjean P: Association of lifetime exposure to fluoride and cognitive functions in Chinese children: a pilot study. Neurotoxicol Teratol 2015, 47:96–101. 25446012 10.1016/j.ntt.2014.11.001

[5] Bashash M, Marchand M, Hu H, Till C, Martinez-Mier EA, Sanchez BN, Basu N, Peterson KE, Green R, Schnaas L : Prenatal fluoride exposure and attention deficit hyperactivity disorder (ADHD) symptoms in children at 6–12years of age in Mexico City. Environ Int 2018, 121(Pt 1):658–666. 30316181 10.1016/j.envint.2018.09.017

[6] Bashash M, Thomas D, Hu H, Martinez-Mier EA, Sanchez BN, Basu N, Peterson KE, Ettinger AS, Wright R, Zhang Z : Prenatal Fluoride Exposure and Cognitive Outcomes in Children at 4 and 6–12 Years of Age in Mexico. Environmental health perspectives 2017, 125(9):097017. 28937959 10.1289/EHP655 PMC5915186

[7] Green R, Lanphear B, Hornung R, Flora D, Martinez-Mier EA, Neufeld R, Ayotte P Muckle G, Till C: Association Between Maternal Fluoride Exposure During Pregnancy and IQ Scores in Offspring in Canada. JAMA Pediatr 2019. 10.1001/jamapediatrics.2019.1729 PMC6704756 31424532

[8] Till C, Green R, Grundy JG, Hornung R, Neufeld R, Martinez-Mier EA, Ayotte P, Muckle G, Lanphear B: Community Water Fluoridation and Urinary Fluoride Concentrations in a National Sample of Pregnant Women in Canada. Environmental health perspectives 2018, 126(10):107001. 30392399 10.1289/EHP3546 PMC6371693

[9] Till C, Green R, Flora D, Hornung R, Martinez-Mier EA, Blazer M, Farmus L, Ayotte P Muckle G, Lanphear B: Fluoride exposure from infant formula and child IQ in a Canadian birth cohort. Environ Int 2020, 134:105315. 31743803 10.1016/j.envint.2019.105315 PMC6913880

[10] Mullenix PJ, Denbesten PK, Schunior A, Kernan WJ: Neurotoxicity of sodium fluoride in rats. Neurotoxicol Teratol 1995, 17(2):169–177. 7760776 10.1016/0892-0362(94)00070-t

[11] Bartos M, Gumilar F, Bras C, Gallegos CE, Giannuzzi L, Cancela LM, Minetti A: Neurobehavioural effects of exposure to fluoride in the earliest stages of rat development. Physiol Behav 2015, 147:205–212. 25921949 10.1016/j.physbeh.2015.04.044

[12] Ferreira MKM, Aragao WAB, Bittencourt LO, Puty B, Dionizio A, Souza MPC, Buzalaf MAR, de Oliveira EH, Crespo-Lopez ME, Lima RR: Fluoride exposure during pregnancy and lactation triggers oxidative stress and molecular changes in hippocampus of offspring rats. Ecotoxicol Environ Saf 2021, 208:111437. 33096359 10.1016/j.ecoenv.2020.111437

[13] Dionizio A, Pereira H, Araujo TT, Sabino-Arias IT, Fernandes MS, Oliveira KA, Raymundo FS, Cestari TM, Nogueira FN, Carvalho RA Effect of Duration of Exposure to Fluoride and Type of Diet on Lipid Parameters and De Novo Lipogenesis. Biological trace element research 2019, 190(1):157–171. 30328034 10.1007/s12011-018-1542-4

[14] Al-Ansari S, Jalali R, Bronckers T, Raber-Durlacher J, Logan R, de Lange J, Rozema F: The effect of a single injection of irinotecan on the development of enamel in the Wistar rats. J Cell Mol Med 2018, 22(3):1501–1506. 29285894 10.1111/jcmm.13415 PMC5824403

[15] Dionizio A, Uyghurturk DA, Melo CGS, Sabino-Arias IT, Araujo TT, Ventura TMS, Perles J, Zanoni JN, Den Besten P Buzalaf MAR: Intestinal changes associated with fluoride exposure in rats: Integrative morphological, proteomic and microbiome analyses. Chemosphere 2021, 273:129607. 33508686 10.1016/j.chemosphere.2021.129607 PMC8076095

[16] Perera T, Ranasinghe S, Alles N, Waduge R: Effect of fluoride on major organs with the different time of exposure in rats. Environ Health Prev Med 2018, 23(1):17. 29769014 10.1186/s12199-018-0707-2 PMC5956923

[17] Dunipace AJ, Brizendine EJ, Zhang W, Wilson ME, Miller LL, Katz BP Warrick JM, Stookey GK: Effect of Aging on Animal Response to Chronic Fluoride Exposure. Journal of dental research 1995, 74(1):358–368. 7876430 10.1177/00220345950740011201

[18] Malin AJ, Bose S, Busgang SA, Gennings C, Thorpy M, Wright RO, Wright RJ, Arora M: Fluoride exposure and sleep patterns among older adolescents in the United States: a cross-sectional study of NHANES 2015–2016. Environ Health 2019, 18(1):106. 31818308 10.1186/s12940-019-0546-7 PMC6902325

[19] Den Besten P Wells CR, Abduweli Uyghurturk D: Fluoride exposure and blood cell markers of inflammation in children and adolescents in the United States: NHANES, 2013–2016. Environmental Health 2022, 21(1):102. 36289513 10.1186/s12940-022-00911-6 PMC9608888

[20] Bai R, Huang Y, Wang F, Guo J: Associations of fluoride exposure with sex steroid hormones among U.S. children and adolescents, NHANES 2013–2016. Environmental Pollution 2020, 260:114003. 31995781 10.1016/j.envpol.2020.114003

[21] Malin AJ, Lesseur C, Busgang SA, Curtin P Wright RO, Sanders AP: Fluoride exposure and kidney and liver function among adolescents in the United States: NHANES, 2013–2016. Environ Int 2019, 132:105012. 31402058 10.1016/j.envint.2019.105012 PMC6754771

[22] Guo M, Afrim F-K, Li Z, Li N, Fu X, Ding L, Feng Z, Yang S, Huang H, Yu F : Association between fluoride exposure and blood pressure in children and adolescents aged 6 to19 years in the United States: NHANES, 2013–2016. International Journal of Environmental Health Research 2023, 33(6):541–551. 35168424 10.1080/09603123.2022.2040449

[23] Abduweli Uyghurturk D, Goin DE, Martinez-Mier EA, Woodruff TJ, DenBesten PK: Maternal and fetal exposures to fluoride during mid-gestation among pregnant women in northern California. Environ Health 2020, 19(1):38. 32248806 10.1186/s12940-020-00581-2 PMC7132865

[24] Madsen EM, Lindegaard ML, Andersen CB, Damm P Nielsen LB: Human placenta secretes apolipoprotein B-100-containing lipoproteins. J Biol Chem 2004, 279(53):55271–55276. 15504742 10.1074/jbc.M411404200

[25] Mackman N: Role of Tissue Factor in Hemostasis, Thrombosis, and Vascular Development. Arteriosclerosis Thrombosis, and Vascular Biology 2004, 24(6):1015–1022. 15117736 10.1161/01.ATV.0000130465.23430.74

[26] Abuhaloob L, Petersen PE: Oral health status among children and adolescents in governmental and private schools of the Palestinian Territories. Int Dent J 2018, 68(2):105–112. 29168573 10.1111/idj.12345 PMC9378908

[27] Bryant JW, Shariat-Madar Z: Human plasma kallikrein-kinin system: physiological and biochemical parameters. Cardiovasc Hematol Agents Med Chem 2009, 7(3):234–250. 19689262 10.2174/187152509789105444 PMC4905712

[28] MacDonald TM, Walker SP Hiscock R, Cannon P Harper A, Murray E, Hui L, Dane K, Middleton A, Kyritsis V : Circulating Delta-like homolog 1 (DLK1) at 36 weeks is correlated with birthweight and is of placental origin. Placenta 2020, 91:24–30. 32174303 10.1016/j.placenta.2020.01.003

[29] Liu XJ, Yuan L, Yang D, Han WN, Li QS, Yang W, Liu QS, Qi JS: Melatonin protects against amyloid-beta-induced impairments of hippocampal LTP and spatial learning in rats. Synapse 2013, 67(9):626–636. 23620224 10.1002/syn.21677

[30] Burton GJ, Fowden AL: The placenta: a multifaceted, transient organ. Philosophical Transactions of the Royal Society B: Biological Sciences 2015, 370(1663):20140066. 10.1098/rstb.2014.0066 PMC4305167 25602070

[31] Shen YW, Taves DR: Fluoride concentrations in the human placenta and maternal and cord blood. Am J Obstet Gynecol 1974, 119(2):205–207. 4823388 10.1016/0002-9378(74)90035-0

[32] Gardner DE, Smith FA, Hodge HC, Overton DE, Feltman R: The Fluoride Content of Placental Tissue as Related to the Fluoride Content of Drinking Water. Science 1952, 115(2982):208–209. 14913209 10.1126/science.115.2982.208

[33] Shen YW, Taves DR: Fluoride concentrations in the human placenta and maternal and cord blood. American Journal of Obstetrics and Gynecology 1974, 119(2) :205–207. 4823388 10.1016/0002-9378(74)90035-0

[34] Ron M, Singer L, Menczel J, Kidroni G: Fluoride concentration in amniotic fluid and fetal cord and maternal plasma. Eur J Obstet Gynecol ReprodBiol 1986, 21(4):213–218. 10.1016/0028-2243(86)90018-3 3709921

[35] Husdan H, Vogl R, Oreopoulos D, Gryfe C, Rapoport A: Serum ionic fluoride: normal range and relationship to age and sex. Clin Chem 1976, 22(11):1884–1888. 975549

[36] Torra M, Rodamilans M, Corbella J: Serum and urine ionic fluoride: normal range in a nonexposed population. Biological trace element research 1998, 63(1):67–71. 9764571 10.1007/BF02785278

[37] Yadav AK, Kaushik CP Haritash AK, Singh B, Raghuvanshi SP Kansal A: Determination of exposure and probable ingestion of fluoride through tea, toothpaste, tobacco and pan masala. Journal of hazardous materials 2007, 142(1–2):77–80. 16979289 10.1016/j.jhazmat.2006.07.051

[38] Going RE, Hsu SC, Pollack RL, Haugh LD: Sugar and fluoride content of various forms of tobacco. J Am Dent Assoc 1980, 100(1):27–33. 6927883 10.14219/jada.archive.1980.0028

[39] Del Carmen AF, Javier FH, Aline CC: Dental fluorosis, fluoride in urine, and nutritional status in adolescent students living in the rural areas of Guanajuato, Mexico. J Int Soc Prev Community Dent 2016, 6(6):517–522. 28032042 10.4103/2231-0762.195510 PMC5184384

[40] Gerlach JC, Thompson RL, Gridelli B, Schmelzer E: Effects of Delta-Like Noncanonical Notch Ligand 1 Expression of Human Fetal Liver Hepatoblasts on Hematopoietic Progenitors. Stem Cells Int 2019, 2019:7916275. 10.1155/2019/7916275 PMC6442310 31011334

[41] Abdallah BM, Ditzel N, Laborda J, Karsenty G, Kassem M: DLK1 Regulates Whole-Body Glucose Metabolism: A Negative Feedback Regulation of the Osteocalcin-Insulin Loop. Diabetes 2015, 64(9):3069–3080. 25918236 10.2337/db14-1642

[42] Ansell PJ, Zhou Y, Schjeide BM, Kerner A, Zhao J, Zhang X, Klibanski A: Regulation of growth hormone expression by Delta-like protein 1 (Dlk1). Mol Cell Endocrinol 2007, 271 (1–2):55–63. 17485162 10.1016/j.mce.2007.04.002 PMC1974851

[43] Hsiao EY, Patterson PH: Placental regulation of maternal-fetal interactions and brain development. Dev Neurobiol 2012, 72(10):1317–1326. 22753006 10.1002/dneu.22045

[44] Azevedo Portilho N, Pelajo-Machado M: Mechanism of hematopoiesis and vasculogenesis in mouse placenta. Placenta 2018, 69:140–145. 29680159 10.1016/j.placenta.2018.04.007

[45] Montalbán-Loro R, Lassi G, Lozano-Ureña A, Perez-Villalba A, Jiménez-Villalba E, Charalambous M, Vallortigara G, Horner AE, Saksida LM, Bussey TJ : Dlk1 dosage regulates hippocampal neurogenesis and cognition. Proceedings of the National Academy of Sciences 2021, 118(11):e2015505118. 10.1073/pnas.2015505118 PMC7980393 33712542

[46] Montalban-Loro R, Lassi G, Lozano-Urena A, Perez-Villalba A, Jimenez-Villalba E, Charalambous M, Vallortigara G, Horner AE, Saksida LM, Bussey TJ : Dlk1 dosage regulates hippocampal neurogenesis and cognition. Proceedings of the National Academy of Sciences of the United States of America 2021, 118(11). 10.1073/pnas.2015505118 PMC7980393 33712542

[47] Giancotti A, Monti M, Nevi L, Safarikia S, D’Ambrosio V, Brunelli R, Pajno C, Corno S, Di Donato V, Musella A : Functions and the Emerging Role of the Foetal Liver into Regenerative Medicine. Cells 2019, 8(8). 10.3390/cells8080914 PMC6721721 31426422

[48] Bonnet A-L, Chaussain C, Broutin I, Rochefort GY, Schrewe H, Gaucher C: From Vascular Smooth Muscle Cells to Folliculogenesis: What About Vasorin? Frontiers in Medicine 2018, 5. 10.3389/fmed.2018.00335 PMC6288187 30564578

[49] Yang L, Cheng X, Shi W, Li H, Zhang Q, Huang S, Huang X, Wen S, Gan J, Liao Z : Vasorin Deletion in C57BL/6J Mice Induces Hepatocyte Autophagy through Glycogen-Mediated mTOR Regulation. Nutrients 2022, 14(17):3600. 36079859 10.3390/nu14173600 PMC9460126

[50] Apostol AC, Jensen KDC, Beaudin AE: Training the Fetal Immune System Through Maternal Inflammation—A Layered Hygiene Hypothesis. Frontiers in Immunology 2020, 11. 10.3389/fimmu.2020.00123 PMC7026678 32117273

[51] Shibata M, Pattabiraman K, Lorente-Galdos B, Andrijevic D, Kim S-K, Kaur N, Muchnik SK, Xing X, Santpere G, Sousa AMM : Regulation of prefrontal patterning and connectivity by retinoic acid. Nature 2021, 598(7881):483–488. 34599305 10.1038/s41586-021-03953-x PMC9018119

